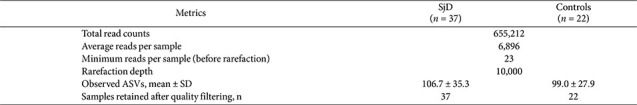# Skin Microbiome Profiling in Patients with Primary Sjögren Disease Compared to Healthy Individuals

**DOI:** 10.4014/jmb.2510.10010

**Published:** 2026-02-11

**Authors:** Sujin Jo, Hoonhee Seo, Kyung-Ann Lee, Sukyung Kim, Md Abdur Rahim, Tapan Indrajeet Barman, Hyun-Sook Kim, Ho-Yeon Song

**Affiliations:** 1 Department of Microbiology and Immunology, School of Medicine, Soonchunhyang University, Cheonan-si 31151, Republic of Korea; 2 Division of Rheumatology, Department of Internal Medicine, Soonchunhyang University Seoul Hospital, Seoul 04401, Republic of Korea; 3 Human Microbiome Medical Research Center (HM ·MRC), Soonchunhyang University, Asan 31538, Republic of Korea

**Keywords:** 16S metagenomics, Primary Sjögren disease, Taxonomic, Biomarker, Skin, Microbiome

## Abstract

Primary Sjögren disease (SjD) is a systemic autoimmune disease characterized by inflammation of exocrine glands, most commonly leading to dry mouth and dry eyes. Although the etiology of SjD remains unclear, emerging evidence suggests that the microbiome modulates immune homeostasis. This study aimed to compare the skin microbiomes of SjD patients with those of healthy controls (HCs) using 16S rRNA gene sequencing. Taxonomic composition, alpha and beta diversity, and predicted functional profiles were evaluated. We observed a significant depletion of *Cutibacterium* and a marked reduction in microbial diversity in SjD patients. Beta diversity analyses revealed distinct clustering among groups. Functional prediction suggested the downregulation of metabolic pathways associated with microbial homeostasis. Our findings propose that alterations in the skin microbiota may contribute to SjD pathogenesis and serve as potential biomarkers or therapeutic targets.

## Introduction

Primary Sjögren’s disease (SjD) is an autoimmune condition that typically develops in middle-aged women and involves chronic, systemic inflammation. It results in immune-mediated damage to exocrine glands, leading to symptoms such as dry mouth (xerostomia) and dry eyes (keratoconjunctivitis sicca) [1]. However, many patients exhibit systemic dryness involving the nasal passages, trachea, vagina, and skin, indicating that the disease extends beyond the exocrine epithelia. Cutaneous involvement is relatively common among these, presenting with various manifestations such as xeroderma, eyelid dermatitis, annular erythema, and cutaneous vasculitis [2].

Recent studies have expanded the scope of SjD research beyond systemic immunity to include the host-associated microbiome [3]. Although many studies have explored the gut microbiome in the context of autoimmune disorders, insights into how the skin microbiome contributes to SjD pathogenesis are still limited. As both a physical and immunological barrier, the skin hosts diverse microbial communities that can influence local and systemic immune responses. A recent pilot study demonstrated that patients with SjD showed significantly elevated erythema index despite no differences in transepidermal water loss or stratum corneum hydration compared with healthy controls (HCs), suggesting subtle but measurable inflammatory skin changes in the absence of overt barrier dysfunction[4]. These findings imply that skin inflammation in SjD may occur independently of classic barrier impairment, potentially driven by immune-mediated or microbiome-mediated mechanisms.

Although the pathogenesis of SjD has traditionally been attributed to adaptive immune mechanisms, such as the activation of Th1/Th17 cells and heightened B cell responses, recent evidence highlights the roles of epithelial apoptosis, impaired autoantigen clearance, and innate immune signaling in disease initiation [5]. Given the close interaction between the skin microbiota and epithelial immunity, it is plausible that skin microbial dysbiosis may contribute to the pathogenesis of SjD, especially by modulating local inflammation or triggering aberrant immune activation.

In this study, we investigated the taxonomic profiles and functional features of the skin microbiota in individuals with SjD, compared with HCs using 16S rRNA gene sequencing.

We focused on taxonomic composition at multiple phylogenetic levels, quantified microbial diversity using alpha and beta diversity metrics, and employed functional prediction tools to assess metabolic pathway differences.

## Materials and Methods

### Study Population

Between June and August 2021, individuals diagnosed with SjD were enrolled in this study. Diagnosis was made according to the 2016 classification criteria proposed by the American College of Rheumatology and the European League Against Rheumatism [6]. The HCs were age- and sex-matched individuals without a history of SjD. HCs were selected to match patients by age and sex and had no prior history of SjD. Exclusion criteria included: being younger than 19 or older than 70; recent (within 3 months) use of antibiotics or probiotic supplements; pre-existing skin conditions such as psoriasis or atopic dermatitis; use of topical steroids or retinoids within 2 weeks before sampling; presence of connective tissue diseases; current pregnancy; or malignancies. Ethical approval was granted by the Institutional Review Board of Soonchunhyang University Seoul Hospital (approval no. 2020-10-021), and all subjects provided written informed consent before participation.

### Clinical and Laboratory Evaluation

Demographic, clinical, and laboratory information were systematically collected for each participant. The diagnostic assessment involved both salivary gland ultrasonography (SGUS) and the EULAR Sjögren’s Syndrome Disease Activity Index (ESSDAI). A single experienced rheumatologist (KAL, with 9 years of expertise in SGUS) conducted all SGUS and ESSDAI evaluations. SGUS was used to identify morphological changes in the major salivary glands associated with SjD [7].

Evaluation of the four primary salivary glands followed the 2019 OMERACT criteria established by the US Outcome Measures in Rheumatology group [8]. This system, designed for use in parotid and submandibular glands in SjD patients, includes four grades: Grade 0 corresponds to a normal parenchyma; Grade 1 reflects slight inhomogeneity without anechoic or hypoechoic regions; Grade 2 indicates moderate inhomogeneity with isolated anechoic/hypoechoic areas surrounded by typical glandular tissue; and Grade 3 represents severe glandular disruption, where diffuse inhomogeneity and widespread anechoic/hypoechoic areas are present without surrounding normal tissue. A score of 2 or greater in the most affected salivary gland was considered indicative of SjD.

ESSDAI was utilized to quantify systemic disease activity. This index assesses involvement across 12 distinct organ domains, each assigned a weighted severity score. Scores below 5 denote low activity, scores between 5 and 13 reflect moderate activity, and values exceeding 13 indicate high systemic disease activity.

Laboratory findings were also recorded, including the presence of anti-Ro/SSA, anti-La/SSB, and anti-centromere antibodies.

### Sample Collection

Skin swab samples were obtained from a standardized site on the volar forearm under aseptic conditions to minimize environmental contamination. Participants were advised not to cleanse the designated skin region or apply any cosmetic products for at least 12 h before sampling. The collection was performed using the NBgene-SKIN (NBG-S22S) kit from Noble Biosciences (Republic of Korea) [9]. Each swab (sterile rayon swab pre-wetted with sterile phosphate-buffered saline) was rubbed firmly across a 4 cm^2^ skin area in a standardized motion (10 strokes horizontally and vertically). The swabs were immediately placed into sterile DNA/RNA-free tubes and stored at –80°C until DNA extraction. They were transported to the Probiotic Microbiome Convergence Center of Soonchunhyang University (Republic of Korea) on dry ice. Negative swabs (opened but not used on the skin) were processed in parallel to monitor for reagent contamination.

### DNA Extraction

Metagenomic DNA was purified from the collected specimens using the QIAamp DNA Mini Kit (Qiagen, Germany) according to the manufacturer’s instructions. The concentration and quality of the isolated DNA were measured using a NanoDrop ND-1000 spectrophotometer (Thermo Fisher Scientific, USA). DNA integrity was additionally verified by agarose gel electrophoresis. All DNA samples were preserved at -20°C until further analyses. Tissue sampling (microdissection), storage, and all experimental procedures were performed under sterile conditions to minimize the risk of contamination. Given that skin is a low-biomass sample and therefore more susceptible to environmental or reagent-derived contamination, we implemented strict contamination-control measures throughout the workflow. Extraction blanks and instrument blanks were included in each batch to monitor potential contaminants, and no detectable DNA was observed in these negative controls. All procedures were performed in a dedicated clean area using sterile, DNA-free consumables.

### Preparation of 16S rRNA Gene Amplicon Libraries

Amplicon libraries for the 16S rRNA gene were prepared from extracted DNA and sequenced using the Illumina iSeq 100 system, following established library preparation guidelines [10]. To target the V4 region of the 16S rRNA gene, primers 515F (5'-GTGCCAGCMGCCGCGGTAA-3') and 926R (5'-CCGTCAATTYYTTTRAGTTT-3') were employed for amplification [11]. PCR was conducted using the 2× KAPA HiFi HotStart ReadyMix (Kapa Biosystems, USA), and the resulting PCR products were cleaned up using AMPure XP beads (Beckman Coulter, UK). For metagenomic sequencing, DNA libraries were constructed using Nextera XT DNA Library Prep Kit (Illumina, USA) according to the manufacturer’s recommendations. Subsequently, the libraries were placed into an iSeq reagent cartridge and sequenced using the Illumina iSeq system.

### Illumina Sequencing and Bioinformatics Analysis of Metagenomics Data

Paired-end reads from sequencing were merged using the FLASH tool, which adjusts short read lengths efficiently [12]. Microbial community data were analyzed via the QIIME platform, a commonly used pipeline for processing microbiome sequencing results [13]. Amplicon sequence variants (ASVs) were inferred using the DADA2 plugin implemented in QIIME2. This approach denoises reads and resolves unique biological sequences with single-nucleotide resolution, avoiding the limitations of OTU clustering. Taxonomic classification of representative sequences was conducted from the phylum to species level based on the Human Microbiome Project database, employing a Bayesian approach with a 97% confidence threshold.[Table T1][Table T2]

Sampling-based OTU analysis assessed bacterial diversity, as shown in the rarefaction curves. Alpha and beta diversity analyses were performed on rarefied datasets (10,000 reads per sample) to normalize sequencing depth; samples with fewer reads were excluded from these analyses. The richness and diversity of bacterial communities were evaluated using α-diversity indices (Chao1, ACE, Simpson, Shannon, and Good’s coverage) [14] . Group comparisons of alpha diversity indices were performed using the non-parametric Mann–Whitney U test, as normality assumptions were not met. .Beta diversity differences were tested using PERMANOVA. Because skin samples are low-biomass and may contain spurious signals introduced during library preparation or sequencing, putative contaminant features were carefully filtered during the bioinformatic workflow. Amplicon sequence variants (ASVs) that appeared exclusively in negative controls or were detected at extremely low relative abundance across samples were removed prior to downstream analyses. Rare taxa present in only a small number of samples or below the minimum abundance threshold were excluded to minimize the influence of background contamination and sequencing noise.

Bacterial community heterogeneity between groups was compared using Student’s t-test. Principal component analysis (PCA) was performed based on unweighted UniFrac distance metrics [15]. Differentially abundant taxa between the two sample types across all taxonomic levels were identified using LEfSe (Linear Discriminant Analysis Effect Size; http://huttenhower.sph.harvard.edu/galaxy/), which also facilitated visualization through taxonomic bar plots and cladograms [16]. Network structures within the bacterial communities were analyzed using the Ecological Network Analysis Pipeline, and the resulting networks were visualized with Cytoscape [17]. Functional profiling of microbial communities between the two groups was predicted using the Phylogenetic Investigation of Communities by Reconstruction of Unobserved States (PICRUSt) algorithm [18]. The MeV (Multiple Experiment Viewer) package was used to perform data clustering and visualization. Bacterial functional profiles were inferred from OTU data using PICRUSt, with reference to Kyoto Encyclopedia of Genes and Genomes (KEGG) database[19]. Operational inferences of the microbiome were generated using PICRUSt2 following guidelines available at https://github.com/picrust/picrust2/wiki. Analysis of variance (ANOVA) was employed to determine significant differences among predicted pathways [20].

## Results

### Baseline Characteristics

A total of 37 female individuals diagnosed with SjD participated in this study. The average duration of illness was 4.1 years (SD ±2.4), and the mean age was 60.7 years (SD ±8.5). Twenty-two healthy subjects were enrolled as controls. All subjects were of Korean nationality. A summary of demographic and clinical baseline characteristics is provided in Table S1. No statistically significant differences in age, sex, or smoking history were found between the SjD and control cohorts. Skin dryness was more frequently reported among SjD patients. More patients with SjD reported skin dryness compared to HCs (64.9% vs. 18.2%, *P* < 0.001). Five SjD patients received treatment: two with hydroxychloroquine monotherapy, two with low-dose prednisone monotherapy, and one with a combination of hydroxychloroquine and low-dose prednisone. None of the patients with SjD were receiving immunosuppressive therapy.

### Taxonomic Composition Analysis

The taxonomic composition of the skin microbiome was assessed at the phylum, class, order, family, and genus levels to compare microbial community structures between SjD patients and HCs.

Across both groups, the predominant bacterial phyla included *Actinobacteria*, *Firmicutes*, *Proteobacteria*, and *Bacteroidetes*. Notably, the relative abundance of *Actinobacteria* was markedly lower in the patient group than in controls, whereas *Proteobacteria* showed a higher proportion in patients. *Firmicutes* levels appeared moderately reduced in the SjD group, whereas *Bacteroidetes* remained relatively consistent between groups.

Consistent with phylum-level findings, the class *Actinobacteria* was substantially depleted in SjD patients. Conversely, Gammaproteobacteria and Alphaproteobacteria, belonging to *Proteobacteria*, were more enriched in the SjD patient group. The class Clostridia, associated with *Firmicutes*, was also slightly elevated in patients, while Bacilli was reduced.

A prominent reduction in Actinomycetales at the order level was observed in SjD patients. In contrast, Pseudomonadales, Enterobacteriales, and Clostridiales were relatively enriched in the patient group. Other orders, such as Lactobacillales and Bacteroidales, showed minor group variations.

Among microbial families, Propionibacteriaceae was significantly reduced in SjD patients, consistent with genus-level depletion of *Cutibacterium*. Increased abundance of Pseudomonadaceae, Enterobacteriaceae, and Neisseriaceae was observed in the SjD group, indicating a shift toward potentially pro-inflammatory or opportunistic taxa. The abundance of families such as Streptococcaceae, Corynebacteriaceae, and Staphylococcaceae remained comparable across groups. At the genus level, the most notable finding was a marked reduction in *Cutibacterium* in the SjD group compared to HCs. This genus, which includes *Cutibacterium acnes*, is a dominant and beneficial skin commensal known for its role in sebum metabolism and maintaining cutaneous immune homeostasis. In contrast, Pseudomonas was relatively increased in the SjD group, suggesting an enrichment of opportunistic bacteria. Other genera, such as *Streptococcus*, *Acinetobacter*, *Staphylococcus*, *Bacteroides*, and *Faecalibacterium*, demonstrated minor shifts in relative abundance between groups but did not display consistent patterns across all participants.

**Fig. 1:**
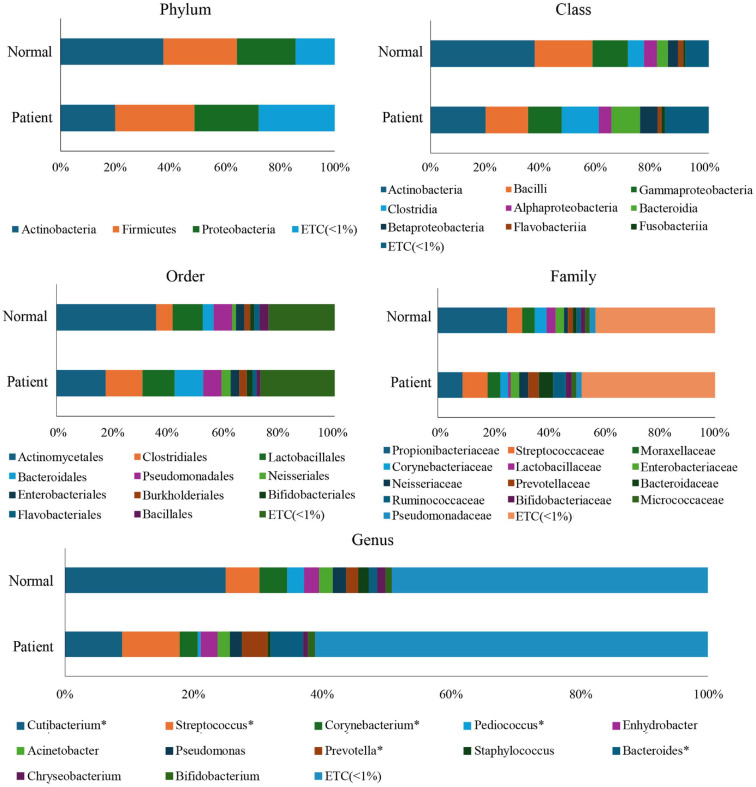
Average taxonomic compositions of healthy controls (HCs) and primary Sjogren’s disease (SjD) groups. The normal group and SjD patients were classified at the phylum, class, order, family, and genus levels. Only the *Cutibacterium* genus showed a significant difference between the two groups among taxa of all ranks. Statistical significance between groups was analyzed using the Wilcoxon rank-sum test. *p < 0.05.

Overall, these taxonomic shifts reflect a dysbiotic state in SjD patient skin microbiota, characterized by a loss of commensal diversity and the emergence of opportunistic taxa, which may contribute to the immune dysregulation observed in SjD pathogenesis. (Fig. 1).

### Alpha Diversity Analysis

Across all metrics, a consistent and significant increase in microbial richness and diversity was observed in SjD patients compared to healthy controls. Specifically, species richness was significantly higher in the SjD group, as indicated by the Observed ASVs count (*p* < 0.01), which reflects the total number of detected species. The Chao1 estimator, which accounts for undetected rare taxa, also showed a notable increase (*p* < 0.01), suggesting an expansion of low-abundance species. Similarly, the ACE index, which considers both common and rare species, was significantly increased in SjD patients (*p* < 0.01), reinforcing the finding of increased microbial richness.

In addition to richness, diversity indices incorporating species evenness revealed significant differences. The Shannon index, which balances species abundance and evenness, was markedly higher in the SjD group (*p* < 0.001), indicating increased community complexity and evenness. The Simpson index, which gives more weight to abundant species, was also significantly increased in patients (*p* < 0.001), suggesting altered community structure and ecological balance. Furthermore, Fisher’s alpha, another measure of diversity, was significantly higher in SjD patients than in HCs (*p* < 0.05).

**Fig. 2:**
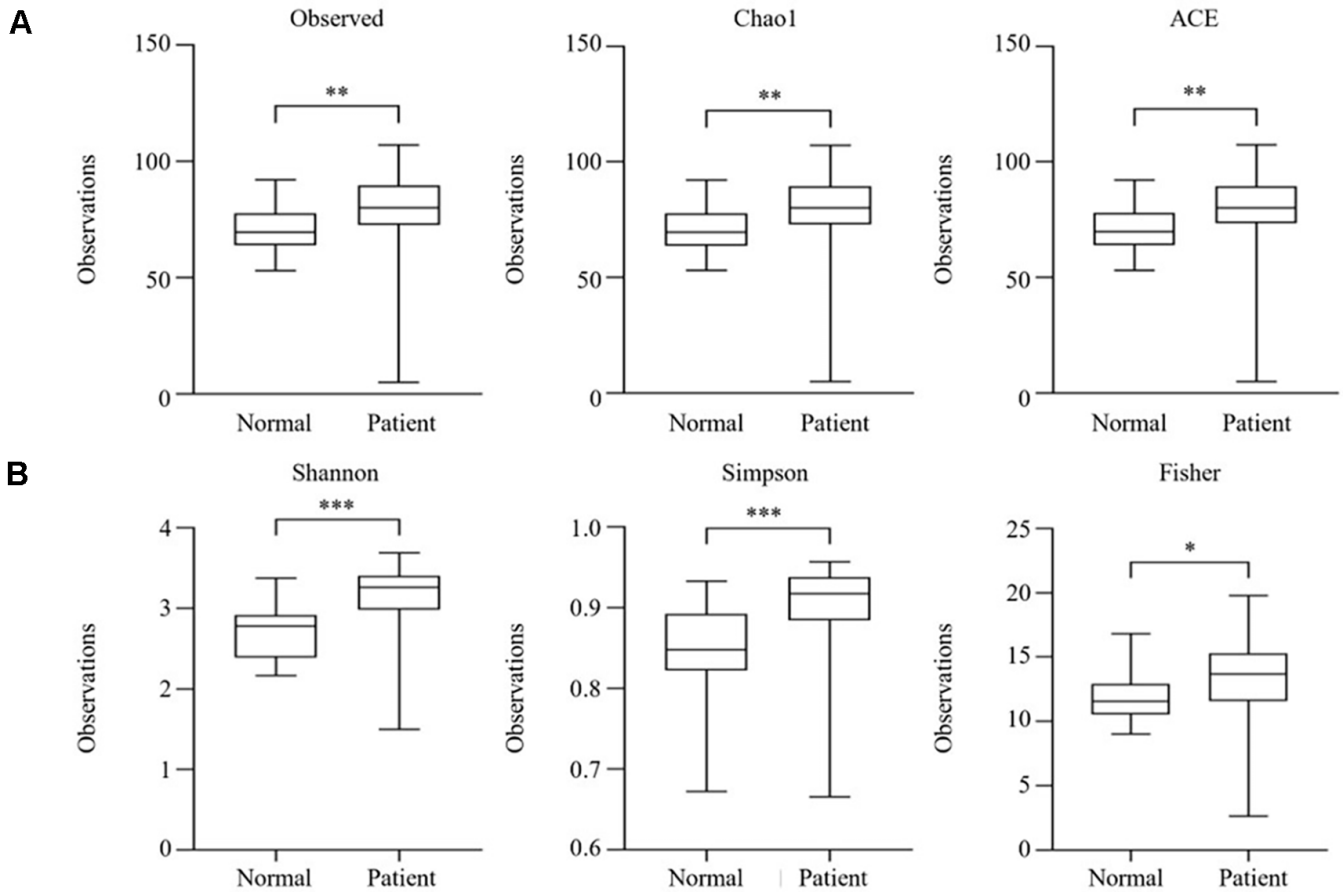
Alpha diversity indices for skin samples of healthy controls (HCs) and patients with primary Sjogren disease (SjD). (**A**) Species richness was analyzed with Observed, Chao1, and ACE, and (**B**) Species diversity was analyzed with Shannon, Simpson, and Fisher. The thick horizontal black band represents the median value, and boxplot margins indicate the first and third quartiles. There was a significant difference between the two groups in the analysis results.

These findings demonstrate that patients with SjD exhibit a significantly more diverse and heterogeneous skin microbiota than HCs. SjD patients exhibited increased microbial richness and evenness compared to healthy controls (Fig. 2).

**Fig. 3:**
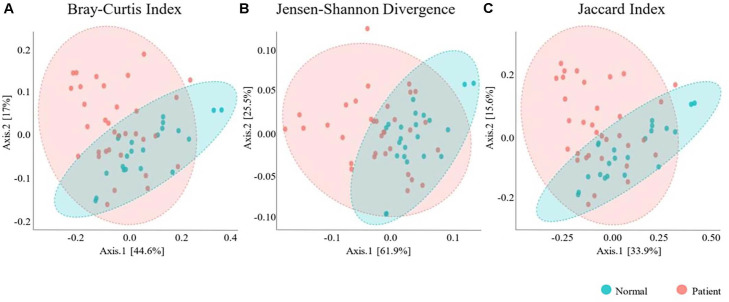
Principal coordinate analysis (PCoA) of bacterial communities. Clustering using the Unweighted Pair Group Method with Arithmetic mean (PERMANOVA). Healthy controls (HCs) and primary Sjogren disease (SjD) patients were analyzed using (**A**) the Bray-Curtis Index, (**B**) the Jensen-Shannon Divergence, and (**C**) the Jaccard Index.

**Beta-diversity analysis revealed a distinct separation between the patient and normal groups.** Principal coordinate analysis (PCoA) based on Bray–Curtis, Jensen–Shannon divergence, and Jaccard indices consistently showed that the microbiome profiles of patients clustered separately from those of normal controls (Fig. 3).

### Hierarchical Clustering Further Supported These Differences in Community Composition

**Fig. 4:**
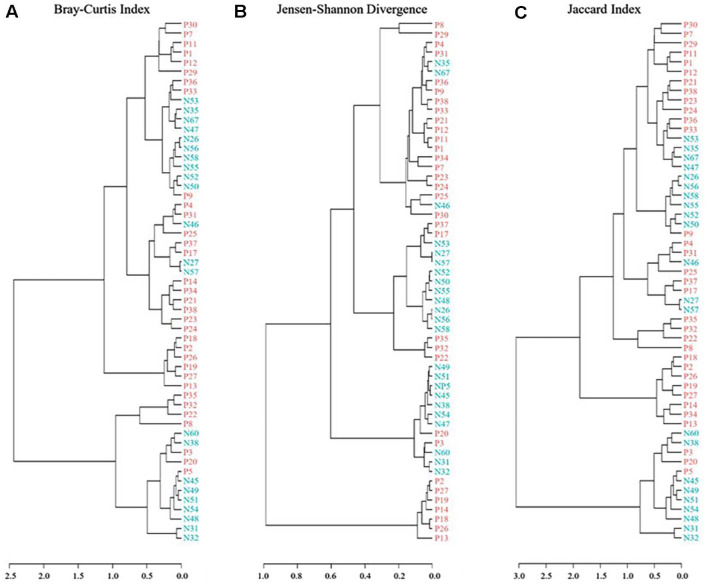
Clustering using the Unweighted Pair Group Method with Arithmetic mean (UPGMA). Healthy controls (HCs) and primary Sjogren’s disease (SjD) were analyzed by (**A**) Bray-Curtis Index, (**B**) Jensen-Shannon Divergence, and (**C**) Jaccard Index.

Dendrograms generated using Bray–Curtis, Jensen–Shannon divergence, and Jaccard distances demonstrated that samples predominantly formed group-specific clusters, indicating distinct structural patterns between the two groups (Fig. 4).

### LDA Score

To identify specific microbial taxa that differentiate the skin microbiome profiles of SjD patients from HCs, we performed LEfSe (Linear Discriminant Analysis Effect Size) analysis. This method enables the detection of statistically significant and biologically relevant features (*e.g.*, taxa) in explaining group differences.

**Fig. 5:**
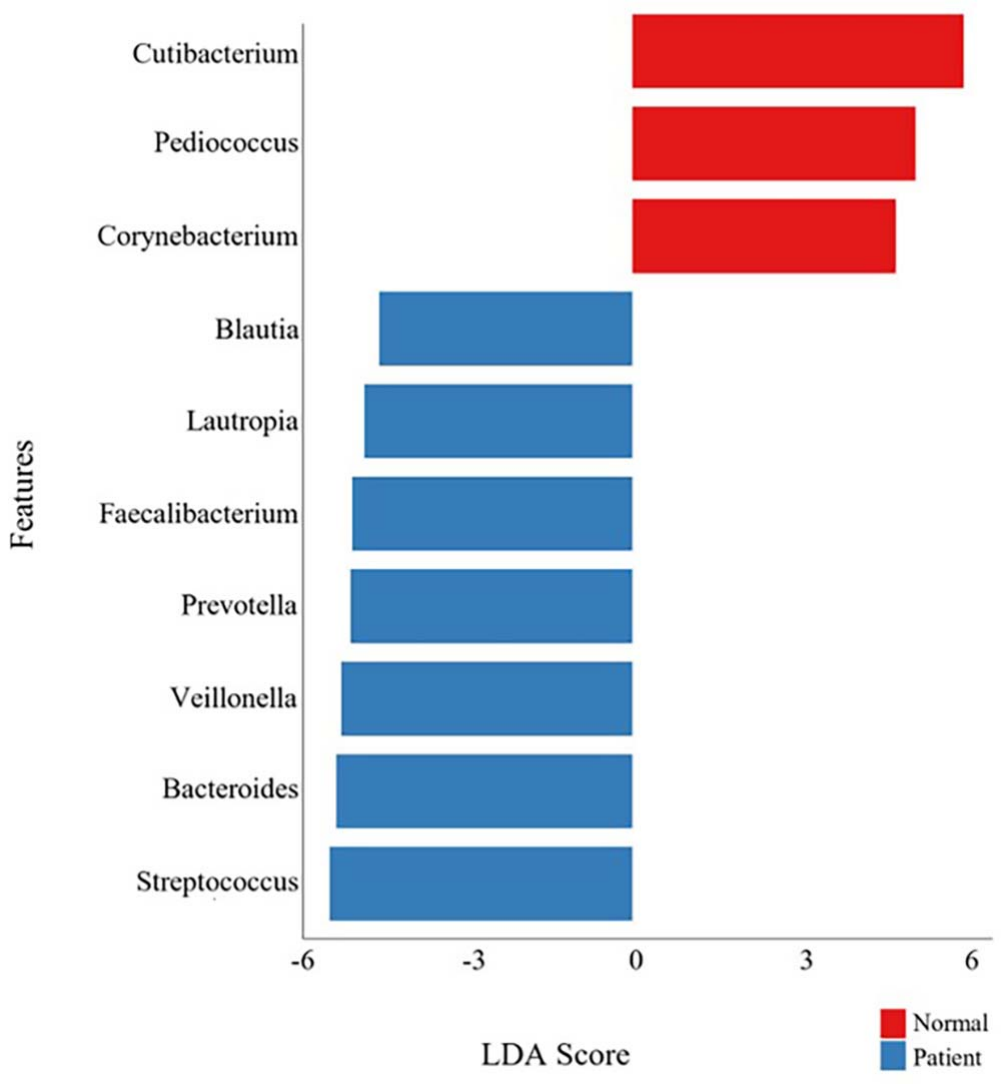
Discriminative bacterial taxa identified using Linear Discriminant Analysis (LDA) Effect Size (LEfSe). Microbial features that significantly differed between skin microbiomes of Healthy controls (HCs) and primary Sjögren’s disease (SjD) patients were identified using LEfSe. Taxa enriched in the normal group included (A) *Cutibacterium*, (B) *Pediococcus*, and (C) *Corynebacterium*, whereas the SjD group showed increased abundance of (D) *Blautia*, (E) *Lautropia*, (F) *Faecalibacterium*, (G) *Prevotella*, (H) *Veillonella*, (I) *Bacteroides*, and (J) *Streptococcus*.

The analysis revealed several genera that were significantly enriched in each group, as reflected in their LDA scores (Fig. 5). In the HCs group, *Cutibacterium*, *Pediococcus*, and *Corynebacterium* were significantly overrepresented and commonly recognized as skin commensals with potential immunomodulatory properties. Notably, now reclassified as *Cutibacterium* had the highest LDA score among all features, supporting its role as a dominant and potentially protective taxon in the healthy skin microbiome.

In contrast, SjD patients exhibited a distinct enrichment of several genera, including *Blautia*, *Lautropia*, *Faecalibacterium*, *Prevotella*, *Veillonella*, *Bacteroides*, and *Streptococcus*. Many of these taxa, such as *Prevotella* and *Veillonella*, are typically associated with mucosal or gut environments, suggesting possible ectopic colonization or altered skin barrier function in SjD. Enrichment of *Faecalibacterium* and *Bacteroides* genera, often linked to systemic immune modulation, may reflect a broader shift in microbial ecology associated with autoimmune dysregulation.

Taken together, these findings highlight distinct microbial signatures in the skin of SjD patients compared with HCs. The observed loss of key commensals and the emergence of atypical or potentially pro-inflammatory taxa may contribute to disease pathogenesis. They could serve as candidate biomarkers for SjD diagnosis or stratification.

### KEGG Path Inference Based on PICRust2

**Fig. 6:**
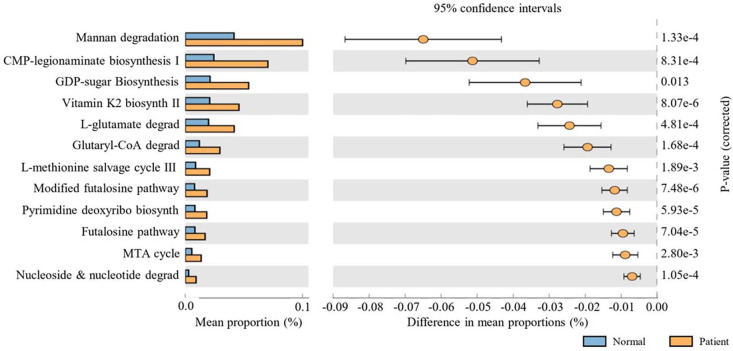
Functional differences between healthy controls (HCs) and primary Sjogren’s disease (SjD) groups. A total of 12 metabolic pathways varied between the two groups. Tests were conducted using the Kyoto Encyclopedia of Genes and Genomes (KEGG), Phylogenetic Investigation of Communities by Reconstruction of Unobserved States (PICRUST), and the MetaCyc webserver. Mannan degradation, CMP-legionaminate biosynthesis I.

To explore potential functional consequences of skin microbiome dysbiosis in SjD, microbial metabolic pathway predictions were generated using PICRUSt2 based on 16S rRNA gene sequences. Differential abundance analysis revealed multiple KEGG pathways significantly underrepresented in the SjD patient group compared to HCs (Fig. 6).

Among the most significantly depleted pathways in SjD patients was mannan degradation (p = 1.33 × 10^-4^), suggesting an impaired capacity of the skin microbiota to degrade complex polysaccharides. This could affect microbial resource competition and host glycan metabolism. Similarly, CMP-legionaminate biosynthesis I (*p* = 8.31 × 10^-4^) and GDP-sugar biosynthesis (*p* = 0.013) were reduced, suggesting potential shifts in microbial glycosylation activities that may influence host–microbe interactions and immune signaling.

Notably, the vitamin K2 biosynthesis II pathway was significantly decreased in SjD patients (*p* = 8.07 × 10^-6^). Microbially produced vitamin K2 contributes to skin and systemic anti-inflammatory effects, and its depletion may exacerbate inflammatory processes in SjD. Likewise, decreased abundance of the L-glutamate degradation and glutaryl-CoA degradation pathways (*p* = 4.81 × 10^-4^ and 1.68 × 10^-3^, respectively) suggests alterations in microbial amino acid metabolism, which may impact local nitrogen cycling and skin pH regulation.

Additionally, the L-methionine salvage cycle III, modified futalosine pathway, and pyrimidine deoxyribonucleotide biosynthesis pathways were significantly less abundant in SjD patients, with corrected p-values ranging from 7.48 × 10^-6^ to 5.93 × 10^-5^. These findings indicate a general reduction in microbial capacity for nucleotide and cofactor recycling. The futalosine pathway and MTA cycle, both associated with menaquinone and methionine turnover, showed significantly reduced representation in the SjD microbiota, indicating further disruption of functional microbial networks.

Together, these results suggest that the skin microbiome in SjD patients is taxonomically altered and functionally impaired, particularly in pathways related to polysaccharide utilization, amino acid and nucleotide metabolism, and anti-inflammatory metabolite production. The loss of these microbial functions may contribute to the chronic inflammatory milieu observed in SjD pathogenesis and represents a potential target for microbiome-based interventions.

### Microbial Diversity and Composition Are Not Significantly Altered by Clinical Parameters in SjD Patients

To investigate the impact of clinical features on the skin microbiome in patients with SjD, we analyzed microbial diversity and composition based on three key clinical parameters: anti-Ro antibody status, SGUS status, and systemic disease activity measured by the ESSDAI. Anti-Ro/SSA antibodies are among the most characteristic autoantibodies in SjD and are associated with earlier onset, higher disease activity, and extra-glandular involvement. SGUS is an imaging modality used to detect structural abnormalities in the major salivary glands, and SGUS positivity (score ≥2) is commonly interpreted as indicative of glandular involvement. ESSDAI is a validated composite index that reflects systemic disease activity across 12 organ-specific domains and is commonly categorized as mild (<5) or moderate-to-high (≥5) activity.

Alpha diversity metrics were compared across patient subgroups, including observed features, Shannon entropy, Faith’s phylogenetic diversity, and evenness. No statistically significant differences were observed in any alpha diversity index between anti-Ro–positive and –negative patients (Fig. S1A), between SGUS-positive and –negative patients (Fig. S2A), or between patients with mild and moderate-to-high ESSDAI scores (Fig. S3A). These findings suggest that the overall richness and diversity of the skin microbiota are not significantly influenced by systemic or glandular disease activity.

Similarly, beta diversity analyses using Bray–Curtis dissimilarity, Jensen–Shannon divergence, and Jaccard index showed no distinct microbial community clustering associated with clinical stratification (Figs. S1B, S2B, S3B). Principal coordinate analysis (PCoA) and hierarchical clustering revealed no distinct clustering pattern by clinical grouping. Additionally, PERMANOVA analysis indicated that microbial community composition did not differ significantly between groups.

Despite the lack of global diversity shifts, differential abundance analyses using LEfSe identified specific microbial taxa that were differentially enriched across clinical subgroups. In anti-Ro–positive patients, Bifidobacterium, *Blautia*, and Oscillospira were enriched, whereas Stenotrophomonas and Methylobacterium were more abundant in the anti-Ro–negative group (Fig. S1C). In SGUS-negative patients, Micrococcus, Sphingomonas, and Lachnospira were enriched (Fig. S2C). Similarly, in patients with mild ESSDAI scores, Actinomyces, Antarctobacter, and Atopobium were identified as potential biomarkers (Fig. S3C).

Together, these findings suggest that while overall skin microbial diversity remains stable across clinical subgroups of SjD, specific taxa may be differentially associated with systemic or glandular disease activity and could serve as candidate microbial indicators of the disease phenotype.

## Discussion

This study provides novel insights into the skin microbiome of patients with SjD, revealing significant shifts in microbial composition, diversity, and functional potential compared to HCs. While prior research has primarily focused on the gut and oral microbiota in SjD, our findings extend this understanding to the skin—a key immunological and barrier organ. Notably, this study builds on our previous work investigating the skin microbiome in systemic sclerosis, in which we also reported depletion of beneficial commensals, such as *Cutibacterium* and *Pediococcus*, and enrichment of anaerobic gram-negative genera associated with disease severity. Similar microbial patterns were observed in the current study of SjD, suggesting possible shared mechanisms of dysbiosis and immune modulation across autoimmune diseases.

In SjD patients, taxonomic analysis revealed a marked depletion of *Cutibacterium* (formerly *Cutibacterium*), a dominant commensal bacterium known for maintaining skin health. *Cutibacterium* contributes to cutaneous immune homeostasis by regulating skin pH, metabolizing lipids, and inhibiting pathogen colonization—particularly in sebaceous areas. Its depletion in SjD patients may reflect a disruption of the skin's lipid environment and barrier integrity, thereby promoting inflammatory conditions. The depletion of this species may reduce the production of microbial-derived immunomodulatory molecules, such as short-chain fatty acids and porphyrins, potentially influencing local and systemic immune responses [21].

Likewise, Pediococcus—a gram-positive lactic acid bacterium (LAB) with antimicrobial, immunomodulatory, and skin-protective properties—was significantly reduced in SjD skin. This genus has been shown to inhibit pathogen growth through bacteriocin production, stimulate anti-inflammatory cytokines (*e.g.*, IL-4 and IL-13), and suppress pro-inflammatory responses (*e.g.*, TNF-alpha) [22]. In our prior study on systemic sclerosis, *Pediococcus* was also diminished in patients and enriched in HCs , suggesting a shared pattern of commensal loss across autoimmune diseases with cutaneous involvement [23]. Additionally, Pediococcus has been implicated in enhancing skin barrier function and reducing transepidermal water loss, highlighting its potential as a therapeutic probiotic candidate in SjD.

Conversely, genera typically associated with mucosal or gut environments—such as Streptococcus, Prevotella, Veillonella, and Bacteroides—were enriched in the skin of SjD patients. These taxa have been implicated in dysbiosis and immune activation in various autoimmune conditions. Their presence on the skin suggests either ectopic colonization or compromised barrier function, possibly reflecting inflammatory skin changes that facilitate colonization by non-resident microbiota.

We evaluated multiple alpha diversity indices reflecting species richness and evenness to explore further differences in skin microbial community structure between the groups. Across multiple alpha diversity indices, SjD patients exhibited significantly higher diversity than HCs. Rather than reflecting a healthier or more stable microbial environment, this increase likely indicates greater microbial heterogeneity driven by the loss of dominant resident commensals and the influx of opportunistic or environmentally derived taxa. Such patterns have been reported in other inflammatory skin conditions, where barrier disruption permits colonization by a wider array of non-resident microorganisms, resulting in elevated but unstable alpha diversity.

Beta diversity analyses further revealed distinct microbial community structures between SjD patients and HCs, supporting the existence of a disease-specific skin microbiome signature. LEfSe analysis identified discriminatory taxa: *Cutibacterium*, *Corynebacterium*, and *Pediococcus* were predominant in controls, whereas SjD samples were enriched in Streptococcus, Bacteroides, Veillonella, and Faecalibacterium—some of which were also associated with severe disease phenotypes in systemic sclerosis.

Functional predictions using PICRUSt2 revealed a significant reduction in microbial metabolic pathways associated with anti-inflammatory activity, glycan metabolism, and epithelial homeostasis in SjD. Specifically, depletion of the mannan degradation pathway suggests reduced microbial capacity for polysaccharide utilization. At the same time, decreased vitamin K2 biosynthesis and methionine salvage cycle indicate impaired production of key anti-inflammatory and redox-regulating metabolites [24]. These functional alterations are supported by recent gut microbiome studies in SjD, which reported reduced microbial diversity, increased pro-inflammatory taxa, and disrupted amino acid and lipid metabolism contributing to epithelial barrier dysfunction and immune dysregulation. Our findings also echo previous observations in systemic sclerosis, where enrichment of gram-negative anaerobes was associated with altered carbon and lipopolysaccharide-related pathways, suggesting a shared pattern of microbial functional dysregulation across autoimmune skin conditions.

Importantly, we found that anti-Ro antibody status, SGUS findings, or systemic disease activity did not significantly influence global microbial diversity and community structure. However, subgroup analyses identified specific microbial taxa associated with clinical phenotypes, suggesting that certain bacteria may serve as microbial correlates or indicators of disease expression without broader compositional shifts.

This study has several limitations. The cohort size was modest and limited to participants at one institution in Korea. Functional profiling was inferred rather than experimentally validated. Moreover, the cross-sectional design does not allow for causal inference. Nonetheless, our results add to a growing body of literature indicating that altered skin microbial ecosystems are a shared feature of systemic autoimmune diseases with cutaneous involvement.

## Conclusion

**Fig. 7:**
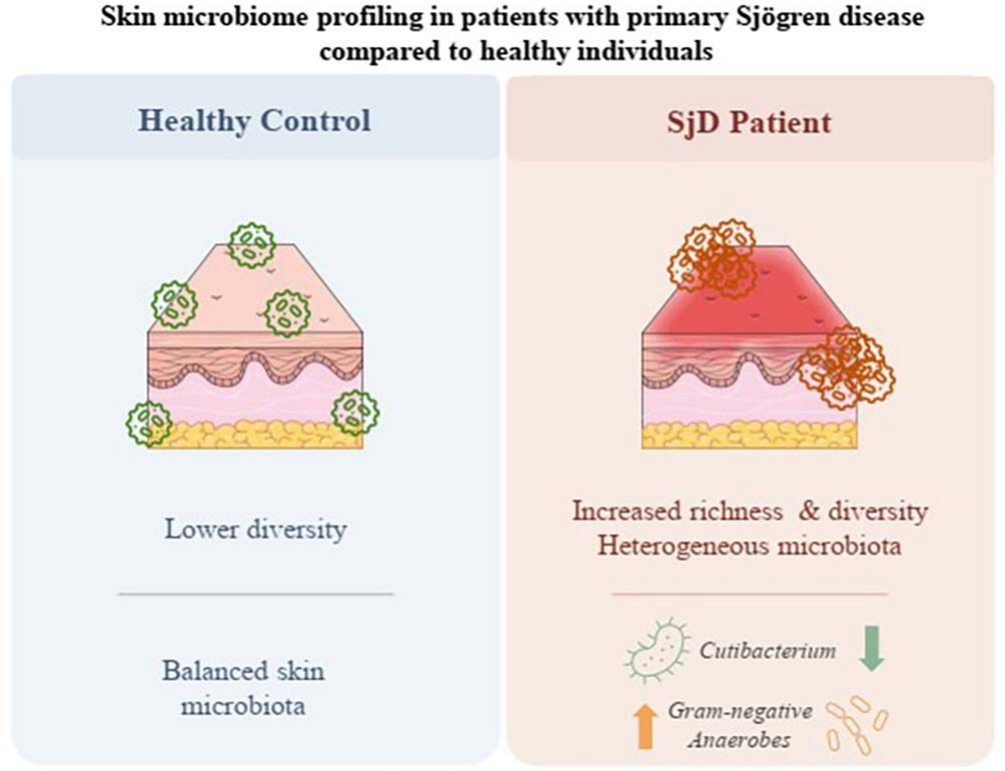
Schematic overview of skin microbiome alterations in patients with primary Sjögren’s disease. This schematic illustrates the key differences in skin microbiome composition between healthy controls and patients with SjD. Compared with healthy controls, who exhibit a balanced skin microbiota with lower diversity, SjD patients show increased microbial richness and diversity, accompanied by a more heterogeneous microbial community structure. In SjD patients, the relative abundance of commensal *Cutibacterium* is reduced, whereas gram-negative anaerobic taxa are relatively increased. These compositional shifts reflect dysbiosis of the skin microbiome associated with SjD.

This study shows that patients with SjD have a disrupted skin microbiome, characterized by reduced microbial diversity, loss of beneficial commensals such as Cutibacterium and Pediococcus, and an increase in potentially pro-inflammatory taxa, including Bacteroides and Streptococcus (Fig. 7). Functional predictions also revealed reductions in anti-inflammatory activity and skin homeostasis pathways, including vitamin K2 biosynthesis and methionine metabolism.

These patterns mirror findings in systemic sclerosis and suggest shared mechanisms of microbiome-associated immune dysregulation in autoimmune skin diseases. The skin microbiome may represent a useful biomarker and therapeutic target in SjD. Further longitudinal studies are needed to clarify causality and explore microbiome-based interventions.

## Supplemental Materials

Supplementary data for this paper are available on-line only at http://jmb.or.kr.



## Figures and Tables

**Table 1 T1:** Baseline characteristics of the study population.

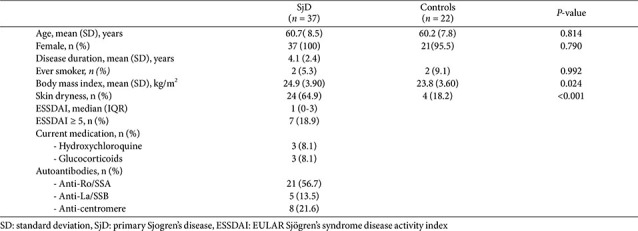

**Table 2 T2:** Sequencing depth and quality metrics of skin microbiome samples.