# Supplementary material for: Skin Microbiome Profiling in Patients with Primary Sjögren Disease Compared to Healthy Individuals
Source: J Microbiol Biotechnol. 2026 Feb 11;36:e2510010. doi: 10.4014/jmb.2510.10010 (PMC12935507; doi:10.4014/jmb.2510.10010)
Supplement: Supplementary file 1 [file jmb-36-e2510010-supple.pdf]

J. Microbiol. Biotechnol. 2026. 36: e2510010
https://doi.org/10.4014/jmb.2510.10010

Table S1. Baseline characteristics of the study population

	**SjD (n=37)**	**Controls (n=22)**	**P-value**
**Age,** mean (SD), years	60.7 (8.5)	60.2 (7.8)	0.814
**Female**, n (%)	37 (100)	21(95.5)	0.790
**Disease duration,** mean (SD), years	4.1 (2.4)		
**Ever smoker,** ***n (%)***	2 (5.3)	2 (9.1)	0.992
**Body mass index,** mean (SD), kg/m^2	24.9 (3.90)	23.8 (3.60)	0.024
**Skin dryness,** n (%)	24 (64.9)	4 (18.2)	<0.001
**ESSDAI**, median (IQR)	1 (0-3)		
**ESSDAI≥5**, n (%)	7 (18.9)		
**Current medication,** n (%)			
- Hydroxychloroquine	3 (8.1)		
- Glucocorticoids	3 (8.1)		
**Autoantibodies, n (%)**			
- Anti-Ro/SSA	21 (56.7)		
- Anti-La/SSB	5 (13.5)		
- Anti-centromere	8 (21.6)		

SD: standard deviation, SjD: primary Sjogren's disease, ESSDAI: EULAR Sjögren's syndrome disease activity index

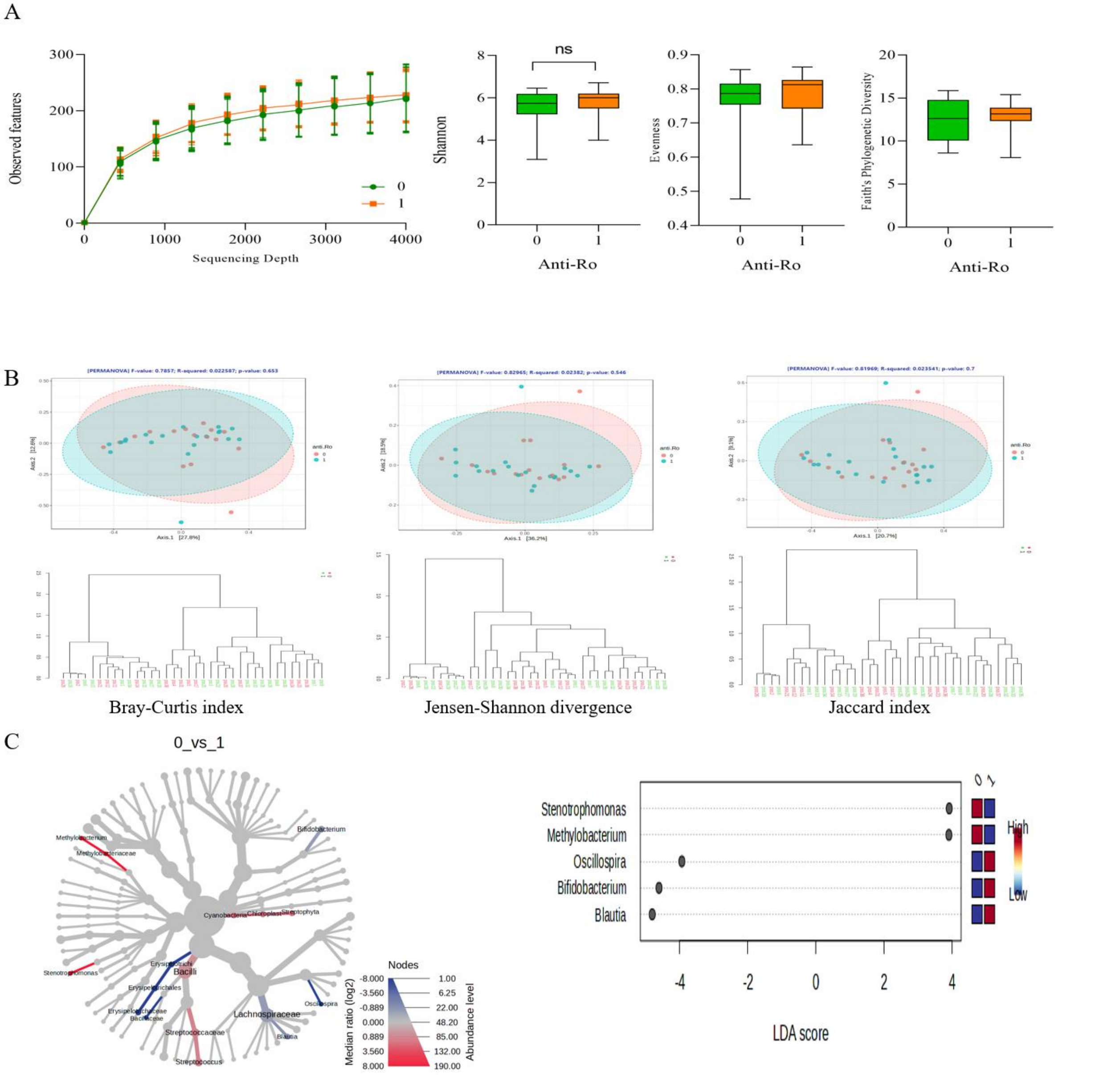

Fig. S1. Effects of anti-Ro antibody status on the skin microbiome of patients with Primary Sjögren's disease. (A) Alpha diversity metrics, including Observed Features, Shannon index, Evenness, and Faith's Phylogenetic Diversity, were compared between anti-Ro–negative and anti-Ro–positive patients. No statistically significant differences were observed across any of the measured indices. **(B)** Beta diversity was assessed using Bray–Curtis dissimilarity, Jensen–Shannon divergence, and the Jaccard index. Principal coordinate analysis (PCoA) and hierarchical clustering revealed no distinct microbial community separation between groups. PERMANOVA analysis confirmed the absence of statistically significant differences in overall microbial composition. **(C)** Differential abundance analysis using LEfSe identified *Blautia, Bifidobacterium,* and *Oscillospira* as enriched in the anti-Ro–positive group, whereas *Stenotrophomonas* and *Methylobacterium* were more abundant in the anti-Ro–negative group. For all panels, group "0" represents the anti-Ro–negative group, and group "1" represents the anti-Ro–positive group.

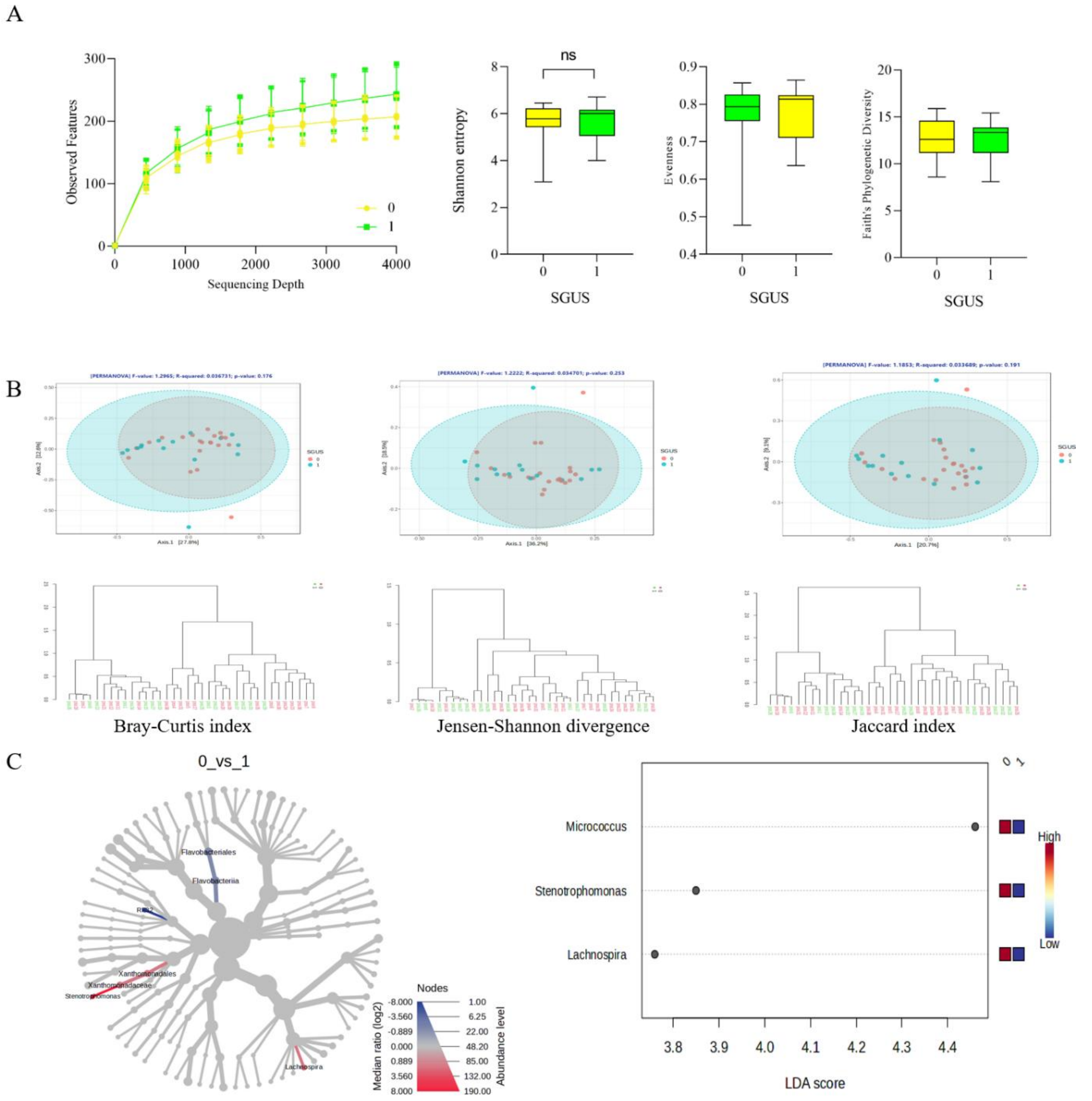

Fig. S2. Effects of salivary gland ultrasonography (SGUS) status on the skin microbiome of patients with Primary Sjögren's syndrome (SjD). (A) Alpha diversity indices including Observed Features, Shannon entropy, Evenness, and Faith's Phylogenetic Diversity were compared between SGUS-negative (score <2) and SGUS-positive (score ≥2) patients. No statistically significant differences were observed across the alpha diversity metrics. **(B)** Beta diversity was assessed using Bray–Curtis dissimilarity, Jensen–Shannon divergence, and the Jaccard index. Principal coordinate analysis (PCoA) and hierarchical clustering revealed no distinct microbial community patterns between SGUS groups. PERMANOVA analysis confirmed the absence of statistically significant differences in microbial community structure. **(C)** Differential abundance analysis using LEfSe identified *Micrococcus, Sphingomonas,* and *Lachnospira* as potential biomarkers enriched in the SGUS-negative group. In all panels, group "0" refers to the SGUS-negative group (score < 2), and group "1" refers to the SGUS-positive group (score ≥ 2).

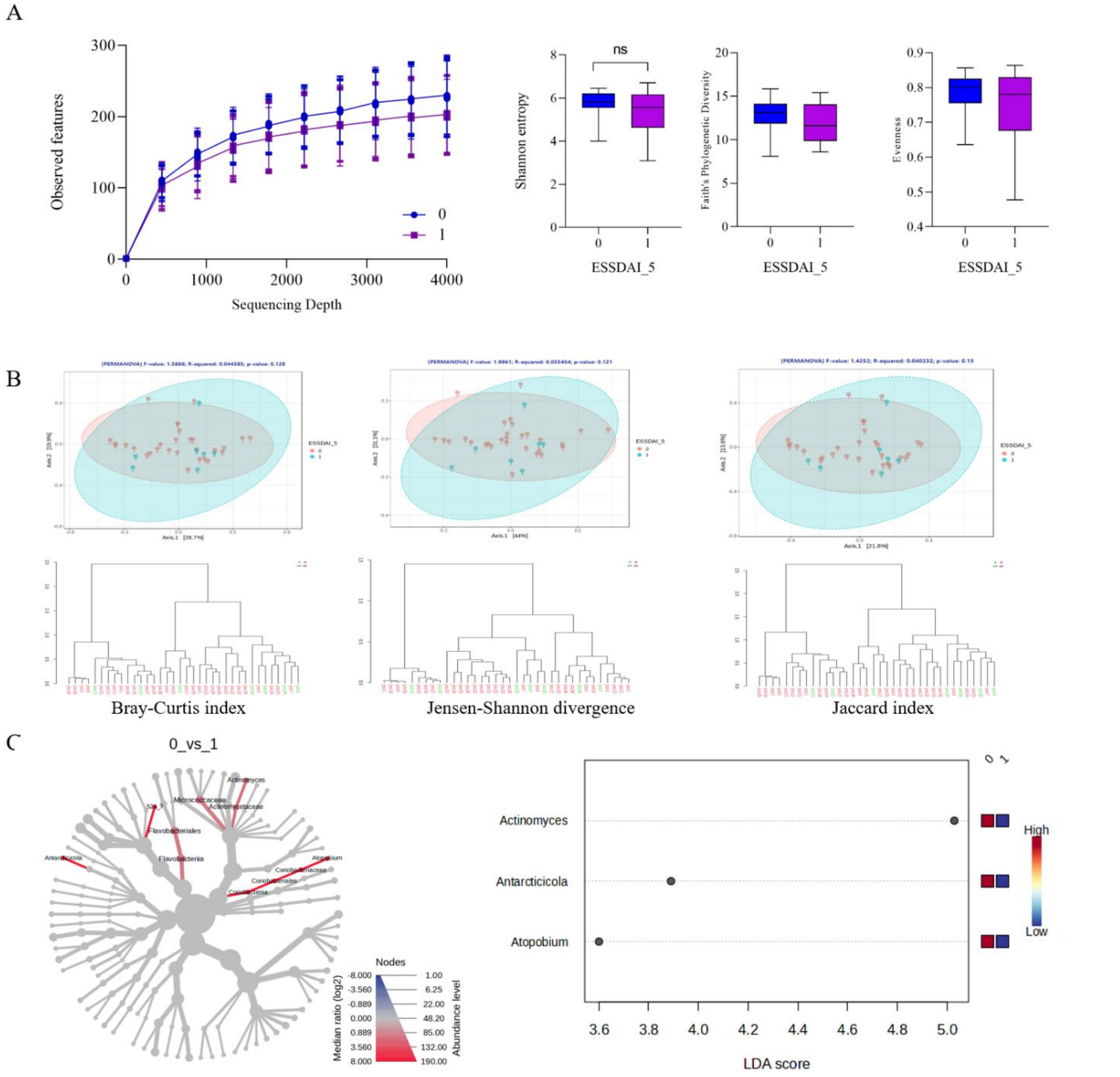

Fig. S3. Systemic disease activity (ESSDAI score) effects on patients with Primary Sjögren's disease (SjD)**'s skin microbiome. (A)** Alpha diversity metrics—including Observed Features, Shannon entropy, Faith's Phylogenetic Diversity, and Evenness—were assessed based on ESSDAI-defined disease activity (mild: ESSDAI < 5; moderate-to-high: ESSDAI ≥ 5). No statistically significant differences were observed across all diversity indices.

(B) Beta diversity was evaluated using Bray–Curtis dissimilarity, Jensen–Shannon divergence, and the Jaccard index. PCoA plots and hierarchical clustering analyses revealed no separation between the mild and moderate-to-high ESSDAI groups. PERMANOVA tests confirmed the absence of statistically significant differences in microbial community structure. **(C)** LEfSe analysis identified *Actinomyces, Antarctobacter,* and *Atopobium* as potential biomarkers enriched in patients with mild disease activity (ESSDAI < 5). In all panels, group "0" refers to patients with mild disease activity (ESSDAI < 5), and group "1" refers to those with moderate-to-high disease activity (ESSDAI ≥ 5).
